# Hemoadsorption efficacy for uncomplicated high-risk cardiac surgery

**DOI:** 10.1186/s13054-019-2629-9

**Published:** 2019-11-04

**Authors:** Sébastien Redant, Matthieu Legrand, Yael Langman, Alejandra Garcia Aguilar, François Angoulvant, Keitiane Kaefer, David De Bels, Rachid Attou, Kianoush Kashani, Patrick M. Honore

**Affiliations:** 1 ICU Department, Centre Hospitalier Universitaire Brugmann-Brugmann University Hospital, Place Van Gehuchtenplein,4, 1020 Brussels, Belgium; 2 Department of Anesthesiology, Critical Care and Burn Unit, St. Louis Hospital, University Paris 7 Denis Diderot, UMR-S942, Inserm, Paris, France; 3 Hospital General de Zona No. 14 IMSS, Universidad Autónoma de Guadalajara, Guadalajara, Mexico; 4 Service d’accueil des Urgences Pédiatriques, Necker-Enfants Malades University Hospital, Assistance Publique–Hôpitaux de Paris, Paris, France; 5 Division of Nephrology and Hypertension, Division of Pulmonary and Critical Care Medicine, Mayo Clinic, Rochester, MN USA

We enthusiastically read the paper by Poli et al. related to the use of CytoSorb® in high-risk cardiac surgery. While use of CytoSorb® showed no clinical or laboratory benefit, it was not associated with any complications either [1]. We noted the low level of interleukin-6 (IL-6) reported in this study (i.e., median values < 50 pg/ml and < 200 pg/ml during and after CBP). This observation may explain why there was no obvious benefit. In the paper by Träger et al., a series of 16 post-cardiac surgery patients with systemic inflammation (SI) characterized by a mixture of cardiogenic shock and distributive shock with acute kidney injury requiring continuous renal replacement therapy were enrolled. The authors observed patients prior to treatment with CytoSorb® to have baseline IL-6 and IL-8 levels between 500 and 10,000 pg/mL and 50 and 1000 pg/mL, respectively. Along with a decrease in the interleukin levels, hemodynamic parameters including cardiac index and mean arterial pressure improved and the need for catecholamine declined [2]. The same benefit was observed when CytoSorb® was used intraoperatively for patients with endocarditis. These patients had a high level of IL-6 and IL-8 prior to the intervention [3]. It is also reported that among septic patients with elevated IL-6 (> 1000 pg/mL) requiring continuous renal replacement therapy, cytokine clearance was more efficient when hemoperfusion and hemoadsorption were utilized [4]. Patients with a significant preoperative SI, such as in endocarditis or sepsis or with complicated surgery, are the most likely to benefit from CytoSorb® therapy. We agree with the authors that some cardiac surgery patients might benefit from hemoadsorption device, but we believe that the indication should be tested in patients with a high level of circulating cytokines—such as IL-6 [5].

## Data Availability

Not applicable.

## Abbreviations

CBP: Cardiopulmonary bypass
IL-6: Interleukin-6
IL-8: Interleukin-8
SI: Systemic inflammation
